# Different interpretation of additional evidence for HTA by the commissioned HTA body and the commissioning decision maker in Germany: whenever IQWiG and Federal Joint Committee disagree

**DOI:** 10.1186/s13561-019-0254-6

**Published:** 2019-12-17

**Authors:** C. M. Dintsios, F. Worm, J. Ruof, M. Herpers

**Affiliations:** 1 Institute for Health Services Research and Health Economics, Medical Faculty, Heinrich Heine University, Building: 12.49 Moorenstr. 5, 40225 Düsseldorf, Germany; 2 Health Economics, University Duisburg-Essen, Essen, Germany; 3 Medical School of Hannover, Hannover, Germany; 4 r-connect ltd, Basel, Switzerland; 5 ClinStat GmbH, Cologne, Germany

**Keywords:** Addenda, AMNOG, IQWiG, Federal Joint Committee, Early benefit assessment, (added benefit, Evidence quality, Agreement statistics), I18

## Abstract

**Background:**

The purpose of this study was to analyse the impact of commissioned addenda by the Federal Joint Committee (FJC) to the HTA body (IQWiG) and their agreement with FJC decisions and to identify potential additional decisive factors of FJC.

**Methods:**

All available relevant documents up to end of 2017 were screened and essential content extracted. Next to descriptive statistics, differences between IQWiG and FJC were tested and explored by agreement statistics (Cohen’s kappa and Fleiss’ kappa) and ordinal logistic regression.

**Results:**

Most of the 90 addenda concerned oncological products. In all contingent comparisons, positive changes in added benefit or evidence level on a subpopulation basis (*n* = 124) prevailed negative ones. Fleiss’ ordinal kappa for agreement of assessments, addenda, and appraisals reached a moderate strength for added benefit (0.474, 95%-CI, 0.408–0.540). Overall agreement between addenda and appraisals on a binary nominal basis is poor for added benefit (Cohen’s kappa 0.183; 95%-CI: 0.010–0.357) ranging from “less than by chance” (respiratory diseases) to “perfect” (neurological diseases). The OR of the selected regression model showed that i) mortality, ii) unmet need, the positions of iii) the physicians’ drug commission and iv) medical societies, and v) the annual therapeutic costs of the appropriate comparative therapy had a high influence on FJC’s appraisals deviating from IQWiG’s addenda recommendation.

**Conclusions:**

IQWiG’s addenda have a high impact on decision-maker’s appraisals offering additional analyses of supplementary evidence submitted by the manufacturers. Nevertheless, the agreement between addenda and appraisals varies, highlighting different decisive factors between IQWiG and FJC.

## Introduction

With the ‘Act to Reorganize the Pharmaceutical Market in the Statutory Health Insurance System’ (AMNOG) pharmaceutical manufacturers have to submit a benefit dossier to the German self-administrative health care decision maker, the Federal Joint Committee (FJC), which effectuates the framework provided by the legislation and ensures that legal instructions are implemented in the healthcare system [1]. FJC commissions the Institute for Quality and Efficiency in Health Care (IQWiG), which was established as a professionally independent, supporting scientific institute. IQWiG primarily prepares evidence reports on pharmaceuticals and non-drug interventions and assesses the Early Benefit Assessment (EBA) dossiers of new pharmaceuticals. For orphan drugs applies a special legal framework, which accounts for the fact that they do not have to prove an added benefit over an appropriate comparative therapy previously determined by the FJC. Their added benefit has already been approved within the granting of an orphan designation by the EMA [2]. The FJC decides only upon the extent of the additional benefit of orphan drugs. The special legal framework is repealed if an orphan drug exceeds a turnover limit of 50 million Euros within 12 months of marketing. In this case, it is reassessed with the same procedure as a non-orphan drug. The methodological basis of the benefit assessment is covered in IQWiG’s publication on ‘General Methods’ [3] and some specific publications [4, 5]. IQWiG’s evaluation results in a recommendation to FJC regarding the added patient-relevant benefit of the investigated pharmaceutical.

**Fig. 1:**
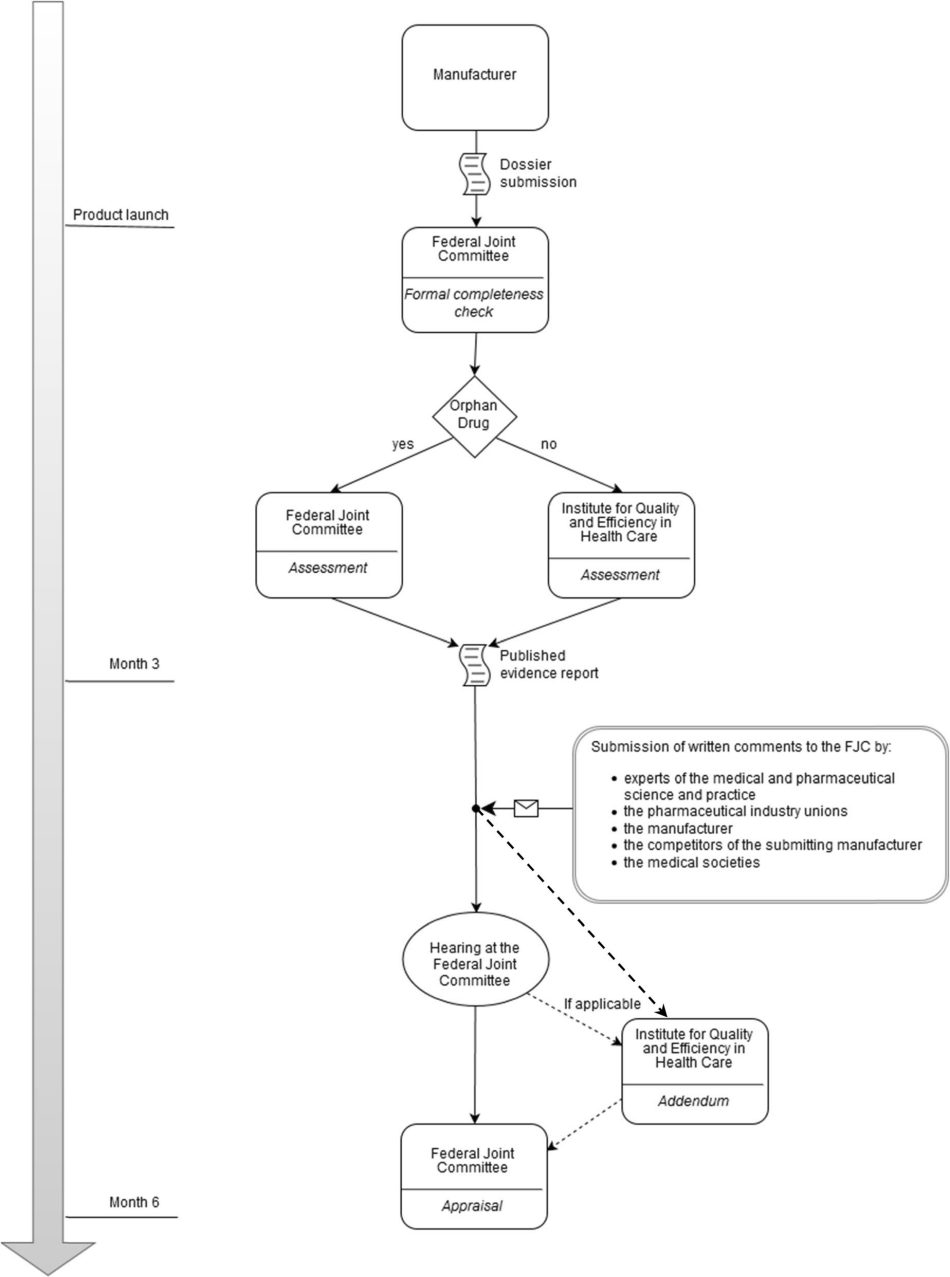
The process of the early benefit assessmentLegend: the figure depicts the AMNOG process in its first step until the final appraisal of the Federal Joint Committee

A hearing is established with regard to submitted comments on IQWiG’s evidence report (assessment) by entitled stakeholders in between the time of recommendation by IQWiG and the time of the final decision by FJC (appraisal). Addenda can be commissioned by FJC in consequence of submitted comments, as a result of the hearing or in cases in which the need for additional work arises during consultations. Addenda offer supplementary information provided at short notice by IQWiG on respective issues. The complete process from dossier submission to the point of appraisal is delineated in Fig. 1.

Outcomes considered for EBA in terms of added benefit are grouped into four dimensions: mortality, morbidity, (severe) adverse events, and health-related quality of life (HRQoL). De facto adverse events can be subsumed to morbidity, since they are usually balanced with morbidity endpoints and IQWiG’s added benefit quantification approach is, depending on severity of the morbidity and adverse events, the same for both dimensions. Fewer adverse events in comparison to the comparator are considered as an added benefit of the assessed pharmaceutical. All available information on adverse events have to be included in the dossier [3, 6].

In the case of an acknowledged added benefit this benefit can vary in different extents (major, considerable, and minor or in the case of a not determinable added benefit: not quantifiable). Further, the benefit can also be classified as not available (no added benefit) or lesser in comparison to the comparator. FJC determines the appropriate comparator and additional subgroups for assessment [6]. The EBA is mainly comparator-driven. In case the submitted evidence misses the defined appropriate comparative therapy (ACT), an added benefit can be derived only by conducting very challenging indirect comparisons for the respective subgroups, if possible [7]. Furthermore, in case of no added benefit granted the ACT sets the price anchor for new drugs. The annual therapeutic costs (AnTC) of the new drug cannot exceed the AnTC of the ACT or it has to be assigned to a reference price group, if any. Missing the ACT has been a common formal reason for denying an added benefit in the past [8] and has lead in some cases to withdrawals of the concerned pharmaceuticals from the German market [9].

In addition, the quality of the evidence base is evaluated. The evidence level is rated as proof, indication, or hint on the basis of the number and characteristics of the submitted studies, the uncertainty of the results, and the consistency of the observed treatment effects [3]. Manufacturers and the SHI negotiate the reimbursement amount of the assessed pharmaceuticals on a subgroup-basis, taking into consideration amongst others the assessment results [10]. If no agreement is reached, an arbitration board is called [11].

A unique phenomenon in the German health policy environment is that the commissioned HTA body (IQWiG) and the commissioning decision maker (FJC) are, contrarily to all other HTA jurisdictions, publishing their own EBA, respectively. This allows exploring differences in the approaches of the involved players when assessing the submitted evidence. Hence, investigating these differences can confirm the assumed impact of addenda on the EBA and lead to a better understanding of the German HTA approach for pharmaceuticals on an international level. Furthermore, it allows involved stakeholders (pharmaceutical companies, medical societies and even the different parties of the decision maker) to draw conclusions on which might be decisive factors within addenda and whether additional data submission by pharmaceutical companies can change IQWiG’s recommendations with a subsequent decision-relevant influence. This part of the EBA has not been investigated so far, even though plenty of national and international publications on AMNOG exist.

The aim of the present work is to describe and to analyse the agreement between IQWiG’s recommendations included in the addenda and FJC’s decision as well as the issues for and the potential impact of commissioned addenda by FJC on its decisions. With the provision of addenda, both, IQWiG and FJC, gain insights into the same submitted latest available evidence, even if theoretically, FJC may also identify evidence itself. Unlike previous publications, that base their comparison of IQWiG and FJC only on the published IQWiG assessments of the evidence in the submitted dossiers and FJC documents [8, 12, 13], any identified discrepancies cannot be justified by different evidence. Furthermore, we aimed at identifying additional potential decisive factors and their impact on FJC appraisal. This exceeds the research questions dealt with in the existing literature on AMNOG. Their analysis relies solely on IQWiG’s assessments. Due to their special legal framework, orphan drugs were not included in the analysis in case they did not exceed the 50 million Euro turnover limit.

## Methods

From the commencement of the AMNOG legislation in January 2011 until end of 2017 every AMNOG procedure including FJC commissioned addenda was studied, critically reviewed and analysed. In order to do so, we proceeded with a multistage approach comprising five steps:

1. All available documents, related to the pharmaceuticals for which FJC commissioned addenda were screened. These were: (i) IQWiG’s assessments, (ii) hearing protocols, (iii) IQWiG’s addenda, (iv) FJC’s decisions, and (v) FJC’s decision rationales.
2. Alongside IQWiG’s addenda structure and the FJC’s decision we developed a spreadsheet for capturing the decisive content. As decisive was defined any derivable information from the screened documents with a direct or potential indirect link to the result of the EBA [extent of added benefit, evidence level, acceptance of endpoints and endpoint quality (mortality versus morbidity inclusively side effects or health-related quality of life), unmet need (available comparable pharmaceuticals in the indication of interest), generic comparator (annual therapeutic costs of the appropriate comparative therapy), potential budget impact (target population size multiplied with annual therapeutic costs of the assessed drug), and position of the influencing stakeholders at the hearing (i.e. medical societies [14] and the German drug commission of the physicians)]. The annual therapeutic costs of the appropriate comparative therapy were included as potential decisive, since there is an ongoing debate on “bad governance” regarding EBA in the sense that SHI being part of the FJC could strategically anticipate the EBA and the subsequent price negotiations by influencing the choice of the comparator [15, 16]. This generated a similar list of essential variables of the EBA as included in previous literature [8, 13]. Thus, all relevant issues of the addenda (i.e. additionally submitted data, relevance of endpoints, bias susceptibility, etc.) were identified, extracted and classified according to their decisive content. Two independent reviewers (CMD, FW) extracted the data. The completed spreadsheets were compared to identify any deviations. Any disagreement was resolved through discussion between the authors.
3. The next step included descriptive statistics of the addenda on a case by case basis as well as on a subgroup basis. The analysis on a subgroup basis reflects more closely the EBA. Gender, age, disease severity, and disease state are the predefined subgroups required. Additional subgroups might be assigned as appropriate to target products to patients who benefit most in accordance with effect modification. Hence, slicing is one of EBA cornerstones [17, 18] even if this is accompanied by substantial power losses [19]. Addenda were classified according to therapeutic indications of medicines. The analysis specifically addressed changes in the extent of additional benefit and the levels of evidence as stated by IQWiG’s assessment and addenda, and the decision by FJC.
4. To compare the recommendations of IQWiG with FJC’s decisions regarding added benefit and evidence quality in the next step a cross-stakeholder analysis on a subgroup level was conducted. Whenever the number of subgroups between an addendum and an appraisal changed, we considered the number of subgroups in the addendum as the basis of comparison for concordance analysis. In addition, we used two established agreement measures: Cohen’s kappa (pair-wise: IQWiG’s assessments versus IQWiG’s addenda and IQWiG’s addenda versus FJC’s appraisals) [20] and Fleiss’ kappa (three raters: IQWiG’s assessments, IQWiG’s addenda and FJC’s appraisals) [21]. We interpreted the results according to the values proposed in statistical literature [22, 23]. Furthermore, to take the ordinal scale of added benefit and quality of evidence into account weighted Cohen’s kappa, where off-diagonal cells contain weights indicating the seriousness of disagreement, were calculated. To keep the analyses more comprehensive, we subsumed the category “lesser benefit” under the category “no added benefit” forming an aggregated category, as the former was assigned to only one subpopulation in the observation period.
5. Finally, ordinal logistic regression analyses were conducted to estimate the impact of identified potential decisive factors on dissent evaluations between IQWiG and FJC based on implemented addenda. The variable to be explained is the difference between IQWiG’s addenda recommendation and the FJC’s appraisal. Ordinal logistic regression model was chosen, since the depending outcome has ordinal features, i.e. more than two categories and the values of each category have a sequential order (i.e. three-level ordinal variable). The estimates of a logistic regression model can be interpreted as the log of the odds, but for the intercept there is no such interpretation. Therefore, we calculated the models without an intercept. To find the most appropriate model, we used a stepwise backwards selection procedure. Starting with the full model, the variable with the highest *p*-value was removed for the next step. The procedure stopped when one of two criteria was met: (i) there are no more non-significant variables left in the model and (ii) the model fit gets worse. To determine the model fit, the Akaike information criterion (AIC) is used. The AIC is based on the log-likelihood but considers the number of variables in the model. Therefore, the AIC can be used to compare the model fit of models with different number of variables. Since we used the procedure only for an exploratory model specification to identify potential significant explanatory variables, we abstained from a split-sample design which would be proper for the identification of predictors.

All analyses were performed using SAS Version 9.4 (SAS Institute, Cary, North Carolina, USA).

Besides the aggregated analysis, some exemplary cases representing different concordance degrees between IQWiG and FJC for addressed issues are described in detail to get an exact impression of the content of IQWiG’s addenda and their potential impact on the subsequent appraisal by FJC.

## Results

### Aggregated analysis

Overall, addenda within EBA were commissioned by FJC and published thereafter by IQWiG up to the end of 2017. With exception of the first year after the introduction of AMNOG, the proportion of addenda exceeded almost one third of all completed EBA over time.

Most EBA for pharmaceuticals in infectious diseases were accompanied by addenda (48%), least in “other” diseases (28%). The overall distribution of addenda by indication area showed the highest number for oncological products followed by pharmaceuticals for metabolic disorders and infectious diseases (Table 1). Regarding the subgroups in the addenda, oncological indications are still leading but the rank between metabolic disorders and infectious diseases reverses.

**Table 1:** Proportion of addenda by indication area

Indications	Early benefit assessments by indication (N)	Addenda for early benefit assessments by indication (N)	Proportion of addenda within indication (%)	Overall proportion by indication (%)	Subgroups in addenda by indication (N)	Overall proportion of subgroups by indication (%)
Oncology	96	40	42%	44%	56	45%
Metabolic disorders	40	16	40%	18%	18	15%
Infectious diseases	25	12	48%	13%	22	18%
Others	39	11	28%	12%	16	13%
Neurology	16	7	44%	8%	7	6%
Respiratory diseases	13	4	31%	5%	5	4%

The most frequent issue provoking addenda commissions was the submission of additional data to FJC with the written comment procedure by the manufacturers, exceeding two thirds of the cases. About one fifth of the addenda were related to endpoints (i.e. their patient-relevance, operationalisation, minimal important clinical differences etc.). Surprisingly, in almost one of ten cases addenda referred to data submitted with the dossier by the manufacturer (i.e. available for assessment), but previously not considered by IQWiG. Imputation for, and amount of missing data (Regorafenib 2013) and bias susceptibility (Belatacept 2015) were each the main issue in one case, respectively.

**Fig. 2:**
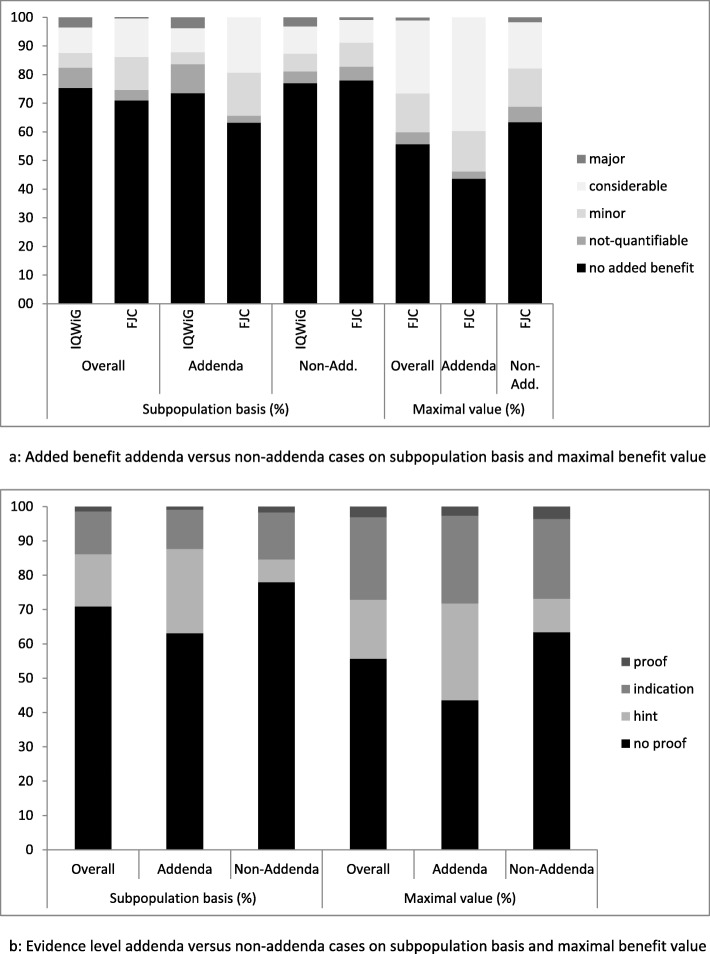
Comparison of IQWiG assessments and FJC appraisalsLegend: the figure compares the results of IQWiG assessments with the appraisals of the Federal Joint Committee divided in addenda and non-addenda cases regarding the extent of added benefit (**a**) and the evidence level (**b**)

Figure 2 contains the results of IQWiG’s assessments and FJC’s appraisals considering extent of added benefit and evidence level, subdivided in cases with and without commissioned addenda for the observed period.

To check for potential differences between cases with and without commissioned addenda, assuming that they would be reflected in base line (assessments) and subsequently in the respective appraisals, IQWiG’s assessments (subpopulation basis), FJC’s appraisals (subpopulation basis and maximal attributed value), and assessments versus appraisals were compared by means of chi-square tests (Table 2). As expected, almost all the comparisons showed statistically significant differences. However, the comparison of IQWiG’s assessments versus FJC’s appraisals on a subpopulation basis for cases with non-commissioned addenda was not statistically significantly different (*p* = 0.347). Surprisingly, the chi-square test for the comparison between cases with and without addenda on a subpopulation basis for added benefit in IQWiG’s assessments came up negative (*p* = 0.117). This is mainly due to the category “no added benefit”. Since almost 85% of this category is ascribed because of formal reasons, there is less chance of differentiation between the cases with and without commissioned addenda with respect to the preceding IQWiG assessment.

**Table 2:** Comparisons between cases with and without addenda for IQWiG assessments and FJC appraisals and between assessments and appraisals

Comparisons	Chi-square	df	*p*-value^a^
Addenda vs non-addenda added benefit on subpopulation basis IQWiG assessments	7.379	4	0.117
Addenda IQWiG assessments vs FJC appraisals added benefit on subpopulation basis	44.287	4	< 10^−5^
Non-addenda IQWiG assessments vs FJC appraisals added benefit on subpopulation basis	4.463	4	0.347
Addenda vs non-addenda added benefit on subpopulation basis FJC appraisals	21.988	4	0.0002
Addenda vs non-addenda evidence level on subpopulation basis FJC appraisals	27.688	3	< 10^−5^
Addenda vs non-addenda maximal added benefit FJC appraisals	14.949	4	0.005
Addenda vs non-addenda maximal evidence level FJC appraisals	12.795	3	0.005
Addenda vs non-addenda maximal added benefit FJC appraisals for all 13 categories^b^	26.733	10	0.003

^a^) alpha = 0.05

^b^) Cat1 “added benefit not proven”

Cat2 “hint of non-quantifiable added benefit”

Cat3 “indication of non-quantifiable added benefit”

Cat4 “proof of non-quantifiable added benefit”

Cat5 “hint of minor added benefit”

Cat6 “indication of minor added benefit”

Cat7 “proof of minor added benefit”

Cat8 “hint of considerable added benefit”

Cat9 “indication of considerable added benefit”

Cat10 “proof of considerable added benefit”

Cat11 “hint of major added benefit”

Cat12 “indication of major added benefit”

Cat13 “proof of major added benefit”

Categories 4 and 13 remained unallocated (df = 10)

**Fig. 3:**
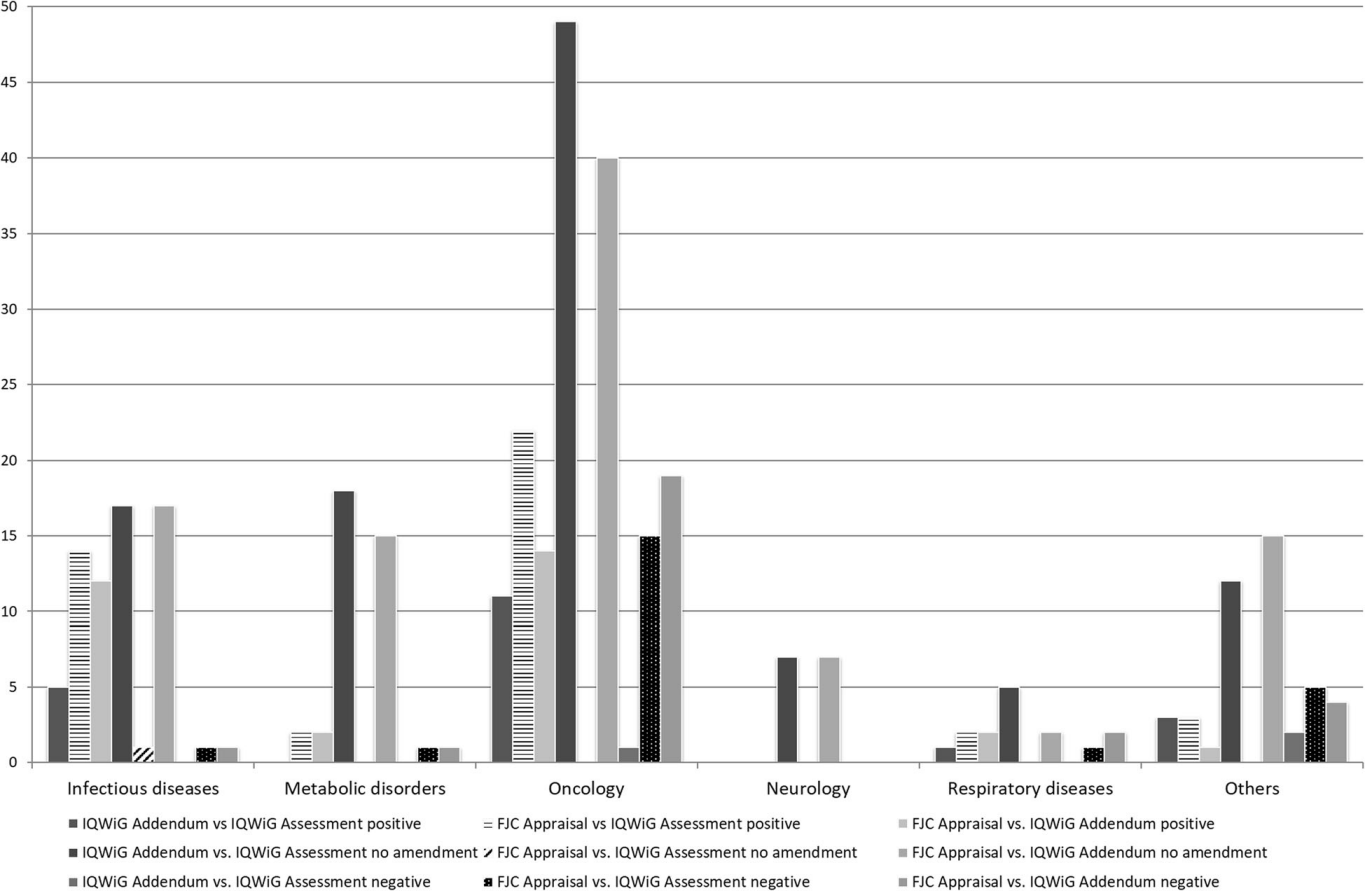
Frequency of changes by specific indicationLegend: the figure visualizes the direction and frequency of the changes of the results comparing the different IQWiG assessments and addenda as well as the appraisals of the Federal Joint Committee with regard to the indication

The frequency analysis of changes regarding any modification in extent of added benefit or level of evidence on a subgroup basis (26 of 90 cases with 60 subgroups) between IQWiG’s assessments, IQWiG’s addenda and FJC’s appraisals showed that in general positive changes prevailed negative changes in all three contingent comparisons. The quantification of added benefit originating from a non-quantifiable added benefit is considered as an improvement. For those pharmaceuticals granted no added benefit, the evidence level is by definition ‘no proof’ and, therefore, any improvement in the benefit level entails an improvement of the evidence level, inevitably. The comparison of FJC’s appraisals versus IQWiG’s assessments reached with 47.8% the highest proportion of changes followed by the comparison of FJC’s appraisals versus IQWiG’s addenda (36.8%) and IQWiG’s addenda versus IQWiG’s assessments (17.6%). This shows that IQWiG stayed with its addenda closer to its preceded assessments than FJC’s appraisals compared to both IQWiG’s assessments and addenda. The latter holds for almost all indications (Fig. 3).

When changes are considered separately with regard to the extent of added benefit and the quality of evidence on a subgroup basis, the positive changes outrange the negative ones for both categories in the comparison between addenda and assessments (Additional file 1: Table S1). Conversely, the comparison of FJC’s appraisals with IQWiG’s addenda shows a more balanced picture between positive and negative changes, suggesting a more heterogeneous pattern.

### Concordance analysis

As shown in Table 3, the overall agreement between IQWiG’s addenda and FJC’s appraisals on a binary nominal basis (down- and upgrades) is poor for the added benefit (Cohen’s kappa 0.183; SE 0.088; 95%-CI: 0.010–0.357) (Table 3b) and fair for the evidence quality (Cohen’s kappa 0.353; SE 0.085; 95%-CI: 0.187–0.520) (Table 3c). The calculated OR for an added benefit FJC versus IQWiG was 2.33 (*p* = 0.028) and for an improvement of the evidence quality 4.53 (*p* <  0.0001), respectively.

**Table 3:** Overall agreement

3a: Contingency table logic					
*Change in level of added benefit or evidence*		Federal Joint Committee			
		+		–	
IQWiG	+	no change (with added benefit)		downgrade by FJC	
	–	upgrade by FJC		no change (no added benefit)	
3b: Cohen’s kappa for the agreement of added benefit IQWiG addenda verus FJC appraisals					
*Level of added benefit*		Federal Joint Committee			
		+		–	
		n	%^b^	n	%^b^
IQWiG	+	23	18.55%	19	15.32%
	–	29	23.39%	53	42.74%
Addenda:	90	Subgroups^a^:	124	*k* = 0.183 (SE: 0.088; CI_95%_: 0.010–0.357)	
Added benefit FJC versus IQWiG: OR = 2.33 (CI_95%_: 1.02–5.36; p = 0.028)					
3c: Cohen’s kappa for the agreement of evidence quality IQWiG addenda versus FJC appraisals					
*Level of evidence*		Federal Joint Committee			
		+		–	
		n	%^b^	n	%^b^
IQWiG	+	32	25.81%	17	13.71%
	–	22	17.74%	53	42.74%
Addenda:	90	Subgroups^a^:	124	*k* = 0.353 (SE: 0.085; CI_95%_: 0.187–0.520)	
Improvement of evidence quality FJC versus IQWiG: OR = 4.53 (CI_95%_:1.96–10.59; p < 0.0001					

*Abbreviation*: Cohens kappa-coefficient (*k*)

^a^26 cases with 60 subpopulations

^b^Proportion of pairs

In Table 4, all results of different agreement statistics for overall as well as for indication specific agreement on benefit extent, evidence level and combined categories of IQWiG’s assessments and addenda, and FJC’s appraisals are depicted.

**Table 4:** Agreement by Cohen’s and Fleiss’ Kappa statistics

Agreement	Kappa	SE/Z^b^	CI95%	Strength of agreement^a^
a. Nominal binary Cohen’s kappa				
Overall addenda vs appraisals added benefit	0.183	0.088	0.010–0.357	poor
Overall addenda vs appraisals evidence level	0.353	0.085	0.187–0.520	fair
Infectious diseases addenda vs appraisals added benefit	−0.029	0.108	−0.241 – 0.183	less than by chance
Infectious diseases addenda vs appraisals evidence level	0.492	0.152	0.194–0.791	moderate
Infectious diseases addenda vs appraisals benefit and evidence level	0.186	0.170	−0.146 – 0.518	poor
Metabolic diseases addenda vs appraisals added benefit	0.308	0.301	−0.283 – 0.898	fair
Metabolic diseases addenda vs appraisals evidence level	0.308	0.301	−0.283 – 0.898	fair
Metabolic diseases addenda vs appraisals benefit and evidence level	0.308	0.301	−0.283 – 0.898	fair
Oncological diseases addenda vs appraisals added benefit	0.073	0.133	−0.188 – 0.333	poor
Oncological diseases addenda vs appraisals evidence level	0.176	0.132	−0.081 – 0.434	poor
Oncological diseases addenda vs appraisals benefit and evidence level	0.188	0.159	− 0.123 – 0.499	poor
Neurological diseases addenda vs appraisals all three outcomes	1.000	1.000	1.000–1.000	perfect
Respiratory diseases addenda vs appraisals added benefit	−0.154	0.415	−0.967 – 0.660	less than by chance
Respiratory diseases addenda vs appraisals evidence level	−0.667	0.248	−1.000 – −0.180	less than by chance
Respiratory diseases addenda vs appraisals benefit and evidence level	−0.500	0.375	−1.000 – 0.235	less than by chance
Other diseases addenda vs appraisals added benefit	0.613	0.199	0.222–1.000	substantial
Other diseases addenda vs appraisals evidence level	0.625	0.181	0.270–0.980	substantial
Other diseases addenda vs appraisals benefit and evidence level	0.800	0.188	0.432–1.000	substantial
b. Ordinal Fleiss’ kappa				
Overall assessments vs addenda vs appraisals added benefit Cat1 “no added benefit” Cat2 “non-quantifiable added benefit” Cat3 “minor added benefit” Cat4 “considerable added benefit” Cat5 “major added benefit”	0.474 0.622 0.384 0.338 0.386 0.375	0.034 Z 12.007 Z 7.399 Z 6.513 Z 7.453 Z 7.229	0.408–0.540	moderate
Overall assessments vs addenda vs appraisals evidence level Cat1 “not proven” Cat2 “hint” Cat3 “indication” Cat4 “proof”	0.520 0.596 0.318 0.638 0.431	0.034 Z 11.505 Z 6.126 Z 12.312 Z 8.306	0.454–0.586	moderate
Overall assessments vs addenda vs appraisals combined categories Cat1 “added benefit not proven” Cat2 “hint of non-quantifiable added benefit” Cat3 “indication of non-quantifiable added benefit” Cat4 “proof of non-quantifiable added benefit” Cat5 “hint of minor added benefit” Cat6 “indication of minor added benefit” Cat7 “proof of minor added benefit” Cat8 “hint of considerable added benefit” Cat9 “indication of considerable added benefit” Cat10 “proof of considerable added benefit” Cat11 “hint of major added benefit” Cat12 “indication of major added benefit” Cat13 “proof of major added benefit”	0.421 0.622 0.241 0.397 NaN 0.293 0.272 0.272 0.191 0.384 −0.005 0.242 0.344 NaN	0.036 Z 12.001 Z 4.649 Z 7.661 NaN Z 5.660 Z 5.246 Z 5.246 Z 3.676 Z 7.401 Z − 0.104 Z 4.665 Z 6.640 NaN	0.351–0.491	moderate
c. Ordinal Cohen’s kappa				
Oncological diseases addenda vs appraisals added benefit Weighted	0.434 0.565	0.091 0.082	0.256–0.611 0.403–0.727	moderate moderate
Without oncological diseases addenda vs appraisals added benefit Weighted	0.456 0.517	0.102 0.095	0.255–0.656 0.331–0.703	moderate moderate
Oncological diseases addenda vs appraisals evidence level Weighted	0.389 0.470	0.098 0.091	0.197–0.580 0.292–0.649	fair moderate
Without oncological diseases addenda vs appraisals evidence level Weighted	0.602 0.733	0.091 0.063	0.423–0.779 0.610–0.857	moderate substantial
Infectious diseases addenda vs appraisals added benefit Weighted	0.314 0.417	0.280 0.249	−0.234 – 0.863 − 0.071 – 0.904	fair moderate
Without infectious diseases addenda vs appraisals added benefit Weighted	0.475 0.582	0.068 0.060	0.342–0.608 0.464–0.700	moderate moderate
Infectious diseases addenda vs appraisals evidence level Weighted	0.824 0.902	0.169 0.094	0.493–1.000 0.717–1.000	almost perfect almost perfect
Without infectious diseases addenda vs appraisals evidence level Weighted	0.488 0.598	0.069 0.057	0.353–0.623 0.486–0.710	moderate moderate
Metabolic diseases addenda vs appraisals added benefit Weighted	0.000 0.000	0.661 0.676	−1.000 – 1.000 −1.000 – 1.000	by chance by chance
Without metabolic diseases addenda vs appraisals added benefit Weighted	0.466 0.526	0.076 0.070	0.317–0.614 0.389–0.664	moderate moderate
Metabolic diseases addenda vs appraisals evidence level (ordinal) Weighted	−0.091 − 0.091	0.568 0.568	−1.000 – 1.000 − 1.000 – 1.000	less than by chance less than by chance
Without metabolic diseases addenda vs appraisals evidence level Weighted	0.598 0.726	0.069 0.049	0.463–0.732 0.631–0.822	moderate substantial

^a^Strength of agreement: < 0 less than by chance, 0.01–0.20 poor, 0.21–0.40 fair, 0.41–0.60 moderate, 0.61–0.80 substantial, 0.81–0.99 almost perfect

^b^if Z exceeds 2.326 there is 99% probability that Kappa > 0; if Z exceeds 1.645 there is 95% probability that Kappa > 0

Regarding the strength of agreement of added benefit between addenda and appraisals the nominal Cohen’s kappa ranges from “less than by chance” (k = − 0.154 respiratory diseases) to “perfect” (k = 1.000 neurological diseases) on an indication specific level but is only for neurological and other diseases (“substantial” agreement) statistically significant. The overall agreement (k = 0.183) seems to be mainly driven by the indications other diseases (k = 0.613) and metabolic diseases (k = 0.308), since neurological diseases are only related to seven cases. Table 4 additionally offers an overview on the strength of agreement of evidence level between addenda and appraisals.

Taking the ordinal character of added benefit and evidence level into consideration and bearing in mind three rating products (IQWiG’s assessments and addenda, and FJC’s appraisals), Fleiss’ kappa reached a moderate strength for added benefit (k = 0.474; SE 0.034; 95%-CI: 0.408–0.540), with all benefit categories exceeding the Z-threshold for a 99% probability that kappa is higher than zero. Similar results were yielded for evidence level (k = 0.520; SE 0.034; 95%-CI: 0454–0.568) and for the combination of added benefit and evidence level (k = 0.421; SE 0.036; 95%-CI: 0.351–0.491)*,* leading to a moderate strength of agreement for both. For the latter, however, no data were available for the categories 4 “proof of non-quantifiable added benefit” and 13 “proof of major added benefit” (unfilled cells), whereas category 10 “proof of considerable added benefit” was the only one with a Fleiss’ kappa less than by chance and a Z-value far below the thresholds for a kappa exceeding zero. This may be due to the fact that this category reflects the highest possible evidence level and the second highest achievable added benefit. The results indicate that there is, with only one exception, a kind of expected agreement even between all three outputs, varying from poor (k = 0.191; category “hint of considerable added benefit” in combined added benefit and evidence level) to substantial (k = 0.638; “indication” in evidence level). The moderate agreement is mainly driven by the category “no added benefit” (“not proven” for evidence level).

In the indication-specific agreement estimation for the three most frequent indications all weighted ordinal Cohen’s kappas were, as expected, higher than the unweighted. For added benefit weighted Cohen’s kappa ranged from “by chance” (weighted k = 0.000; SE 0.676; 95%-CI: − 1.000 – 1.000) in metabolic diseases to “moderate” (k = 0.565; SE 0.082; 95%-CI: 0.403–0.727) in oncological diseases. Only in metabolic diseases the agreement differed compared to the complement (without metabolic diseases) strongly (weighted kappa 0.000 versus 0.526) even though on a nominal binary level they had shown a fair agreement (kappa = 0.308). This indicates a much more heterogeneous valuation between addenda and appraisal, i.e. between IQWiG and FJC, especially for oral antidiabetics.

### Regression analysis

The selection procedure reduced the full model containing all potential influencing decisive factors to five variables (Additional file 2: Table S2). (i) Mortality as endpoint, (ii) need for therapy in that indication, (iii) difference of the German drug commission of the physicians (GDCP) position compared to IQWiG’s recommendation, (iv) difference of the medical societies (MedSoc) positions compared to IQWiG’s recommendation and (v) the annual therapeutic costs of the appropriate comparative therapy (AnTC ACT) were selected. Exemplarily, the calculation of AnTC for Palbocilcib are presented in Additional file 3: Box S1. The selection stopped due to no significant variables left in the model. The overview over the selection process shows that the AIC got smaller with each selection step, indicating a better model fit with each non-significant variable being removed. As additional control, we included McFaddens r-squared, which gives the likelihood-ratio of the current model compared to a model without any covariates and can be interpreted as the amount of variation explained by the current model. The loss in the r-squared is very low and by removing the non-significant variables only 3% is lost. The amount of variation explained by the final model is still at a good 37.5%. Although this value indicates a well-fitted model, it also shows that a high percentage of variation is still unexplained.

The OR of the selected model show that all variables, except AnTC ACT have a high influence on the odds of the FJC deviating from the IQWiG recommendation, although they have a high variation (Table 5). AnTC ACT seems to have only small influence on the odds but, depending on the total costs, the influence can be much higher since the value indicates a difference of every 1.000€. Furthermore, the OR for mortality shows that whenever IQWiG considered mortality endpoints for its addendum recommendation (in most cases (*n* = 22) this led to a high added benefit due to IQWiG’s endpoint-specific algorithmic approach), the FJC tended to downgrade this added benefit (*n* = 10). Hence, consideration of mortality endpoints in IQWiG’s addenda is associated with benefit downgrading by FJC.

**Table 5:** Odd ratios of ordinal logistic regression model

Odds Ratio Estimates				
Effect		Point Estimate	95% Wald Confidence Limits	
Mortality	No versus Yes	3.902	1.474	10.325
Unmet Need	No versus Yes	0.181	0.065	0.505
GDCP	Upgrade versus Downgrade	0.123	0.022	0.689
GDCP	Unchanged versus Downgrade	0.381	0.085	1.717
MedSoc	Upgrade versus Downgrade	< 0.001	< 0.001	0.013
MedSoc	Unchanged versus Downgrade	< 0.001	< 0.001	0.011
AnTC of ACT (per 1000 €)		1.017	1.005	1.030
AIC: 206.050		McFadden R2: 0.37455		

*AIC* akaike information criterion, *AnTC of ACT* annual therapeutic costs of the appropriate comparative therapy, *GDCP* German drug commission of the physicians position, *MedSoc* Medical Scocieties position

### Exemplary cases: addenda for Ticagrelor, Belatacept, and Ixekizumab

Three exemplary cases covering different conditions are presented in detail in the supplement. Ticagrelor for the prevention of atherothrombotic events after myocardial infarction serves as an example for analysis of additional submitted evidence on patient-relevant endpoints and for downgrading the IQWiG’s recommendation on added benefit within its own addendum [24–27]. IQWiG suggested in its addendum that in summary there is no proof of added benefit for Ticagrelor and deviated thereby from its preceded dossier assessment, which resulted in an indication of a minor added benefit. Based on the exact same evidence, FJC granted Ticagrelor in the appraisal a hint for a minor added benefit. Belatacept in the prophylaxis of graft rejection in adults receiving a renal transplant offers an example for commissioning IQWiG with an addendum to investigate potential bias susceptibility after additionally submitted data by the manufacturer [28–31]. FJC appraised Belatacept in concordance with IQWiG, granting an indication of considerable added benefit. Finally, Ixekizumab for the treatment of adult patients with moderate to severe plaque psoriasis is an example for commissioning IQWiG to assess already available data in the value dossier after clarification of the meaning of “pre-treatment” (subpopulation a) and the relevance of an endpoint within the oral hearing of submitted comments to FJC (subpopulation b) [32–35]. IQWiG suggested in its addendum an indication of considerable added benefit, whereas in its preceded assessment [32] after exclusion of respective data it had recommended no added benefit for these patients (subpopulation a). As for the endpoint of interest, a statistically significant difference in favour of Ixekizumab in patients whose nails were found to be affected at the start of the study was shown (subpopulation b). In the subsequent appraisal, FJC granted an indication for a considerable benefit for subpopulation a, in concordance with the IQWiG’s addendum suggestion, and contrary to IQWiG an indication for a minor benefit for subpopulation b [34, 35].

Furthermore, IQWiG sometimes forges methodological paths within addenda preparation not included in its general methods. Exemplarily the following cases are mentioned: the consideration of sample size next to variability of control arms for adjusted indirect comparisons to avoid underestimation of variance (Aflibercept) [36], the requirement of consistency of the results of different (Parkinson-specific) morbidity scales to be accepted (Opicapone) [37] or the differentiation of HRQoL in serious/severe and non-serious/non-severe (Ceritinib) [38].

## Discussion

Whereas submitting additional evidence within HTA processes is internationally rather common, the specific German condition of splitting the assessment and the appraisal with each being assigned to different responsible stakeholders, unlike almost all other European jurisdictions at least, makes it unique. Therefore, to survey effective HTA of pharmaceuticals within the framework of the neo-corporatist governed German health care system from a comparative HTA perspective requires an investigation of both. The agreement between IQWiG’s addenda recommendations and FJC’s decision is in contrast to past comparisons, which referred only to IQWiG’s assessments and FJC’s appraisals [8, 12, 13, 19] the appropriate approach to compare the judgements of the commissioned institute with the commissioning institution, since with the provision of addenda, IQWiG and FJC gain insights into exactly the same latest available evidence basis. Previous publications ignored that IQWiG changed in almost one-fifth its own recommendation within prepared addenda. The reason for commissioning addenda is mainly the evaluation of additional evidence submitted by the manufacturers within the commenting and hearing process after the first assessment by IQWiG, with the assumption that thereby the evidence basis becomes more robust.

Surprisingly, the difference in added benefit between the recommendations of IQWiG for addenda and non-addenda cases showed only a strong numerical trend. On a subpopulation level, this was mainly due to the frequently represented category “no added benefit” because of formal reasons. Hence, the variance of judgements for the remaining subpopulations was not discriminatory enough to reach statistical significance. Furthermore, this result indicates that with regard to the quantification of added benefit the algorithmic approach of IQWiG dominates the assessments and is rather insensitive to potential uncertainties leading to addenda commissions by FJC. As expected, all other tested differences were significant whereas for the non-addenda cases concordance between IQWiG’s assessment and FJC’s appraisal was respectively high. This supports the hypothesis that in these cases IQWiG’s recommendations and FJC’s appraisals are much closer together.

Based on the indication area, most addenda were prepared for oncological products followed by metabolic disorders and infectious diseases. The perfect agreement in nominal Cohen’s kappa for the low number of cases in neurological diseases raises the question, if by its addenda commission FJC sought only an additional rationale for its negative appraisal, since for almost all of these cases no added benefit was granted. The moderate agreement in weighted ordinal Cohen’s kappa for oncological pharmaceuticals reflects the heterogeneous picture of EBA in oncology, where FJC seems to be more pragmatic than IQWiG, in the sense that it is not following a rigid endpoint-based added benefit algorithm and unmet need plays therefore a more important role in comparison to IQWiG’ addenda recommendations. Finally, for oral antidiabetics the respective agreement by chance depicts the extreme rigidity in IQWiG’s addenda with regard to study design and implementation of interventions and comparators. FJC again seems to be somehow more pragmatic in its appraisal highlighting a different decisive approach at least for those oral antidiabetics for which an addendum was commissioned.

With its appraisals, FJC seems to act as a corrective of the IQWiG’s assessments and addenda by wiping out some of the outliers (major and no added benefit) in the distribution of IQWiG’s added benefit recommendations. This can be best described as a “smoothing phenomenon” and is much more pronounced in the addenda cases, as shown by the concordance analysis and the effect of mortality in the ordinal logistic regression as a predictor for downgrading the added benefit recommended by IQWiG’s addenda. On the other hand, FJC’s appraisals for addenda and non-addenda cases on a maximal value basis of the added benefit appear to differ. This indicates a higher proportion of maximal achieved added benefit in the case of commissioned addenda. IQWiG is quantifying added benefit within rigorous assumptions implementing a methodologically strongly criticized mortality-centred algorithmic endpoint-specific approach [19, 39–41] and subsequently proceeding semi-quantitatively to an overall conclusion by balancing negative and positive effects [4]. Then again, FJC only quantifies the added benefit for orphan drugs with all their related restrictions (study design, endpoints, and small collectives) without defining an ACT and following its own methodological approach. This approach unfortunately is not explicitly stipulated in any available document and, therefore, does not allow any reliable conclusions by analogy on potential methodological differences in EBA between FJC and IQWiG, as the latter is not involved in the quantification of added benefit of orphan drugs. Besides the different methodological approaches between IQWiG and FJC, with the latter not applying any sort of known algorithms but acting more context-depending introducing implicitly further and potentially broader decisive criteria than IQWiG, and rather appraising case by case and trying to keep consistency as high as possible, the semi-quantitative overall conclusion is dominated inevitably by value judgements. Thus, FJC already rejected the subpopulation-specific overall conclusions within IQWiG’s first assessment claiming that value judgements have to be legitimated and, therefore, being solely reserved for the decision-maker.

The ordinal logistic regression revealed that potential budget impact had similarly to [13] no significant effect on the added benefit. On the contrary, the annual therapeutic costs of the ACT were significant, showing for every 1.000 € a slight negative impact on the assigned added benefit by the FJC in comparison to IQWiG’s addenda recommendation. Cost considerations of the ACT may influence FJC’s appraisals anticipating the subsequent price negotiations between manufacturers and statutory health insurance and, thereby, being an indicator for bad governance, as the statutory health insurance is next to the medical service providers (physicians, dentists, and hospitals) a constituting stakeholder of the FJC. If one follows this line of argument, it may lead to the conclusion that, whenever the FJC is not able to set a generic ACT, the added benefit is compared to IQWiG’s recommendation downgraded. Nevertheless, it remains speculative if pecuniary motivations explicitly impact downgrading of added benefit in indications with expensive ACT regarding subsequent price negotiations since expensive ACT usually reflect innovative pharmaceuticals (i.e. innovation shifts in the respective indications) reducing the chance for a big added benefit increment in comparison to generic ACT. In the end, the choice of the ACT, the main driver of price negotiations next to the extent of added benefit, was never subject of addenda commissions.

Considering that 7% of the addenda referred to data already submitted by the pharmaceutical manufacturers with their dossier but not included in the IQWiG assessment, the relevance of these data either became obvious within the commenting process and subsequent hearing. Or else FJC, contrary to IQWiG, estimated the value and impact of these data irrespective of the commenting process and hearing as high for the assessment and final appraisal.

Being published at the time of decision-making, addenda commissioned to and prepared by IQWiG are neither subject of a public debate within a commenting process nor allowing any reliable interaction between IQWiG and manufacturers during the short preparation time of less than one and a half month. Consequently, the manufacturers only serve as data provider. With regard to their quality, there is no robust rationale why addenda should be error-free since IQWiG is applying its own methods, just as is does with its evidence reports, when preparing addenda.

The concordance analysis with Cohen’s kappa in terms of inter-rater reliability is applied usually to check for reliability and robustness of the results. We used Cohen’s kappa arbitrarily as an agreement tool, being aware of the different objectives IQWiG (assessment in form of an evidence report culminating in a recommendation) and FJC (appraisal of the decision-maker accompanied by a respective decision rationale) pursue. Yet, this approach has been implemented also by other authors to derive agreement between IQWiG and FJC [13] or different HTA bodies with respect to their assessment outcome [42] or between European Medicines Agency (EMA) and IQWiG [43]. Moreover, we implemented in contrast to [13] next to Cohen’s kappa weighted Cohen’s kappa and Fleiss’ kappa for the strength of agreement and expanded our analysis by means of ordinal logistic regression to identify predictors of difference in added benefit. Unlike [13] (substantial agreement on added benefit with a Cohen’s k 0.64; 95%-CI: 0.451–0.827) the kappa was poor in our analysis (0.183; 95%-CI: 0.010–0.357) indicating the impact of a different comparison approach (IQWiG’s addenda versus IQWiG’s assessments as the reference basis for the agreement with FJC’s appraisals).

Finally, the proposed values for the interpretation of Cohen’s kappa by Altmann [22] and other authors [23] are only arbitrary levels. Due to the generic controversy [44, 45] and further methodological objectives [46] surrounding Cohen’s kappa, results of concordance analysis have to be interpreted cautious. On the other hand, concordance analysis offers much more and in depth information than simple descriptive agreement. Therefore, we present agreement matrixes next to the k-coefficients as proposed by Grouven et al. [47].

Based on a non-systematic selected small sample in [48], the author concluded that the diverging assessments are not always scientifically justifiable, but rather appear to be influenced by the not always transparent framework parameters of the respective health system. Assessments are always shaped by certain perspectives on the data and results under scrutiny. It would undoubtedly be worthwhile to evaluate these influences to gain a better understanding of the reasons for national and international discrepancies in the assessment of additional therapeutic value of new pharmaceutical products [48]. For example, in UK significant unmet need is characterised by a high QALY loss when there is no effective treatment [49], and thereby captured by cost-utility analysis within HTA. In France also the public health benefit of medicines is considered [50]. This was the purpose with our regression on a national level. Based on overall data of the addenda cases we were able to find a model that explains to a fair degree which factors might implicate a difference between FJC’s appraisal and IQWiG’s addenda recommendation. A logistic regression model can only display odds, the lowest ranking measure of association, but due to the nature of the outcome variable, no other model could be applied. The model fit statistics show that the selected model is a good but a high degree of variance in the data is still unexplained. This may be due to the arbitrary nature of the FJC’s decision process or due to some variables not identified. Further research on the factors influencing the deviation between FJC’s appraisal and IQWiG’s recommendation may be necessary. In the past, there were some attempts to identify important decision-making criteria for HTA by logistic regression (see for example [51]). Despite methodological similarities, the HTA approaches differ regarding their focus depending on each jurisdiction (cost-effectiveness analysis versus relative effectiveness assessment [52]), and therefore, results cannot be transferred easily from one jurisdiction to another, especially with regard to the aforementioned German peculiarities.

To our knowledge, this is the first in depth analysis of IQWiG’s addenda and the motivation of FJC to commission IQWiG with the preparation of addenda. Addenda are the appropriate source for the comparison between IQWiGs’ recommendations and FJCs’ appraisals since they are based on the same evidence. Furthermore, this comparison allows within an indication-specific approach, particularly on a subpopulation level, the identification of more and different decisive factors than in the past analyses, as shown in the regression model. In absence of a published FJC specific appraisal approach, our analysis offers more robust results compared to the past comparisons (IQWiG assessments vs FJC appraisals) with regard to additional potential decisive factors.

On the other hand, only publicly available material was included in our analysis. We had no access to confidential decision-making meeting protocols of the FJC nor to FJC (early) advices. Therefore, potentially hidden agendas were not detectable with our approach. Our analysis covers six years (2011–2017). It could be enriched by further addenda cases, published after 2017. Concordance statistics are difficult to interpret regarding drawing conclusions and even our regression model explains a good proportion of variance, there is plenty of room for further investigation.

Manufacturers should take into consideration that additionally submitted data within the commenting and hearing process have a high impact on the EBA, especially with regard to the FJC appraisal and less regarding the addenda themselves, being much closer to the preceded IQWiG assessment recommendations. Whereas the predictability of IQWiG assessments and addenda is somehow due to IQWiG’s endpoint-specific algorithmic approach for the quantification of added benefit higher than that of the FJCs appraisals, there is an opportunity for upgrading IQWiGs’ recommendations because of additional FJC decisive factors. On the other hand, in case of convincing mortality data the algorithmic approach of IQWiG leads to a higher extent of added benefit, which is often downgraded by FJC within its smoothening, but not marginalizing added benefit, approach.

From FJC perspective, additional data add to the beforehand submitted evidence body more usable data. These additional data can impact the quantification of added benefit in two ways: (i) allow for an added benefit and (ii) up- or even downgrade added benefit with regard to IQWiG’s assessment and addenda recommendations. Since additional data can be submitted during the EBA, the process becomes more efficient as it avoids re-evaluations within subsequent new EBA.

## Conclusion

IQWiG’s addenda have a high impact on decision-maker’s appraisals offering additional analyses of supplementary evidence submitted by the manufacturers mainly within the commenting and hearing process of the preceded assessments. Almost in one fifth of the subpopulations of addenda accompanied cases regarding non-orphan drugs IQWiG changed its recommendations. Nevertheless, the agreement between IQWiG’s addenda and FJC’s appraisals on evidence quality and extent of added benefit varies from less than by chance to substantial, depending on the therapeutic indication area – even though they are based on the same submitted evidence. Regarding IQWiG’s recommendations, FJC’s appraisals induce a “smoothing phenomenon” for non-orphan drugs. The agreement analysis and the ordinal logistic regression highlight different decisive factors of the commissioned institute and the commissioning institution. Whenever German EBA is looked at, to compare HTA body and decision maker, addenda have to be considered.

## Supplementary information

**Additional file 1: Table S1.** Overall changes of added benefit and evidence level.

**Additional file 2: Table S2.** Full model

**Additional file 3: Box S1.** Palbociclib AnTC Cost Calculation.

## Data Availability

All data are publicly available and the respective sources are stated in the methods part and in the references.

## Abbreviations

AIC: Akaike information criterion
AMNOG: Act to Reorganize the Pharmaceutical Market in the SHI System
AnTC ACT: Annual therapeutic costs of the appropriate comparative therapy
CI: Confidence interval
EBA: Early benefit assessment
EMA: European Medicines Agency
FJC: Federal Joint Committee
GDCP: German drug commission of the physicians
HRQoL: Health-related quality of life
IQWiG: Institute for Quality and Efficiency in Health Care
MedSoc: Medical societies
NAPSI: Nail psoriasis severity index
OR: Odds Ratio
SE: Standard error
SHI: Statutory health insurance
