# Supplementary material for: Different interpretation of additional evidence for HTA by the commissioned HTA body and the commissioning decision maker in Germany: whenever IQWiG and Federal Joint Committee disagree
Source: Health Econ Rev. 2019 Dec 17;9:35. doi: 10.1186/s13561-019-0254-6 (PMC6918554; doi:10.1186/s13561-019-0254-6)
Supplement: Supplementary file 1 — Additional file 1: Table S1. Overall changes of added benefit and evidence level. [file 13561_2019_254_MOESM1_ESM.docx]

Additional file 1 Table S1: Overall changes of added benefit and evidence level

| IQWiG Addendum vs. IQWiG Assessment | | | FJC Appraisal vs. IQWiG Addendum | |
| --- | --- | --- | --- | --- |
| Change | extent of additional benefit | quality of evidence | extent of additional benefit | quality of evidence |
| Positive | 19 | 15 | 32 | 24 |
| No change | 102 | 107 | 77 | 86 |
| Negative | 3 | 2 | 15 | 14 |
| Sum* | 124 | 124 | 124 | 124 |
| * with subpopulations (26 cases with 60 subpopulations on addendum basis) | | | | |
