# Supplementary material for: Different interpretation of additional evidence for HTA by the commissioned HTA body and the commissioning decision maker in Germany: whenever IQWiG and Federal Joint Committee disagree
Source: Health Econ Rev. 2019 Dec 17;9:35. doi: 10.1186/s13561-019-0254-6 (PMC6918554; doi:10.1186/s13561-019-0254-6)
Supplement: Supplementary file 2 — Additional file 2: Table S2. Full model [file 13561_2019_254_MOESM2_ESM.docx]

Additional file 2 Table S2: Full model

| **Analysis of Maximum Likelihood Estimates** | | | | | | |
| --- | --- | --- | --- | --- | --- | --- |
| **Variable** |  | **DF** | **Estimate** | **Standard Error** | **Wald Chi-Square** | **Pr > Chi²** |
| **Intercept** | **invariant** | 1 | 3.9195 | 0.4315 | 82.5079 | <.0001 |
| **Indication** | **Infectious diseases** | 1 | -1.3033 | 0.5243 | 6.1804 | 0.0129 |
|  | **Metabolic disorders** | 1 | 0.2287 | 0.5349 | 0.1828 | 0.6690 |
|  | **Neurology** | 1 | 0.7407 | 0.7254 | 1.0425 | 0.3072 |
|  | **Oncology** | 1 | 0.0524 | 0.5048 | 0.0108 | 0.9173 |
|  | **Others** | 1 | 0.6333 | 0.5465 | 1.3429 | 0.2465 |
| **Mortality** | **No** | 1 | -0.6938 | 0.3048 | 5.1810 | 0.0228 |
| **Add on therapy** | **No** | 1 | 0.0583 | 0.2608 | 0.0500 | 0.8230 |
| **Need** | **No** | 1 | 0.7839 | 0.2890 | 7.3566 | 0.0067 |
| **GDCP** | **Improvement** | 1 | -0.8813 | 0.4425 | 3.9667 | 0.0464 |
| **GDCP** | **Unchanged** | 1 | 0.0129 | 0.3555 | 0.0013 | 0.9710 |
| **MedSoc** | **Improvement** | 1 | -1.9365 | 0.6391 | 9.1808 | 0.0024 |
| **MedSoc** | **Unchanged** | 1 | -2.6139 | 0.7829 | 11.1487 | 0.0008 |
| **RCT** | **No** | 1 | 0.3257 | 0.2905 | 1.2567 | 0.2623 |
| **Prevalence** |  | 1 | 2.436E-8 | 2.953E-7 | 0.0068 | 0.9343 |
| **AnTC Pharm** |  | 1 | -0.00068 | 0.00344 | 0.0397 | 0.8421 |
| **AnTC ACT** |  | 1 | 0.0164 | 0.00817 | 4.0317 | 0.0447 |
| **Potential BI** |  | 1 | -616E-13 | 4.98E-10 | 0.0153 | 0.9015 |
| AnTC ACT: annual therapeutic costs of the appropriate comparative therapy (comparator)  AnTC Pharm: annual therapeutic costs of the assessed pharmaceutical  DF: number of degrees of freedom  GDCP: German drug commission of the physicians  MSc: Medical Societies  Potential BI: potential budget impact defined as (AnTC Pharm-AnTC ACT)*Prevalence | | | | | | |
