# Supplementary material for: Different interpretation of additional evidence for HTA by the commissioned HTA body and the commissioning decision maker in Germany: whenever IQWiG and Federal Joint Committee disagree
Source: Health Econ Rev. 2019 Dec 17;9:35. doi: 10.1186/s13561-019-0254-6 (PMC6918554; doi:10.1186/s13561-019-0254-6)
Supplement: Supplementary file 3 — Additional file 3: Box S1. Palbociclib AnTC Cost Calculation. [file 13561_2019_254_MOESM3_ESM.docx]

Additional file 3 Box S1: Palbociclib AnTC Cost Calculation

Indication: HR-positive, HER2-negative locally advanced or metastatic breast cancer

(i) Total target population N_total_ = 42555

(ii) Weighted according to subpopulation size using means of cost ranges

(iii) Subpopulation (1): post-menopausal women in first therapeutic treatment line N = 20985

(iv) Annual therapeutic costs (AnTC) = 66,817 € - 77,029 €

(v) Subpopulation-specific AnTC_1_ = 20985/42555 * ((66817+77029)/2) = 35,467 €

(vi) Subpopulation (2): peri-menopausal women in first line treatment N = 3475

(vii) Annual therapeutic costs (AnTC) = 66,817 € - 77,029 € + additional costs = 1,759 € - 2,236 €

(viii) Subpopulation-specific AnTC_2_ = 3475/42555 * ((66817+77029+1759+2236)/2) = 6,036 €

(ix) Subpopulation (3): post-menopausal women with progress after therapy N = 15525

(x) Annual therapeutic costs (AnTC) = 71 € - 54,157 €

(xi) Subpopulation-specific AnTC_3_ = 15525/42555 * ((71+54157)/2) = 9,892 €

(xii) Subpopulation (4): peri-menopausal women with progress after therapy N = 2570

(xiii) Annual therapeutic costs (AnTC) = 71 € - 5409 €

(xiv) Subpopulation-specific AnTC_4_ = 2570/42555 * ((71+5409)/2) = 165 €

(xv) Sum over weighted proportions = 51,560 €
